# Impact of hypertension on coronary artery plaques and FFR-CT in type 2 diabetes mellitus patients: evaluation utilizing artificial intelligence processed coronary computed tomography angiography

**DOI:** 10.3389/frai.2024.1446640

**Published:** 2024-10-23

**Authors:** Yan Xi, Yi Xu, Zheng Shu

**Affiliations:** Shanghai TCM-Integrated Hospital, Shanghai University of Traditional Chinese Medicine, Shanghai, China

**Keywords:** diabetes, hypertension, coronary computed tomography angiography, coronary artery disease, artificial intelligence

## Abstract

**Objective:**

This study utilized artificial intelligence (AI) to quantify coronary computed tomography angiography (CCTA) images, aiming to compare plaque characteristics and CT-derived fractional flow reserve (FFR-CT) in type 2 diabetes mellitus (T2DM) patients with or without hypertension (HTN).

**Methods:**

A retrospective analysis was conducted on 1,151 patients with suspected coronary artery disease who underwent CCTA at a single center. Patients were grouped into T2DM (*n* = 133), HTN (*n* = 442), T2DM (HTN+) (*n* = 256), and control (*n* = 320). AI assessed various CCTA parameters, including plaque components, high-risk plaques (HRPs), FFR-CT, severity of coronary stenosis using Coronary Artery Disease Reporting and Data System 2.0 (CAD-RADS 2.0), segment involvement score (SIS), and segment stenosis score (SSS). Statistical analysis compared these parameters among groups.

**Results:**

The T2DM (HTN+) group had the highest plaque volume and length, SIS, SSS, and CAD-RADS 2.0 classification. In the T2DM group, 54.0% of the plaque volume was noncalcified and 46.0% was calcified, while in the HTN group, these values were 24.0 and 76.0%, respectively. The T2DM (HTN+) group had more calcified plaques (35.7% noncalcified, 64.3% calcified) than the T2DM group. The average necrotic core volume was 4.25 mm^3^ in the T2DM group and 5.23 mm^3^ in the T2DM (HTN+) group, with no significant difference (*p* > 0.05). HRPs were more prevalent in both T2DM and T2DM (HTN+) compared to HTN and control groups (*p* < 0.05). The T2DM (HTN+) group had a higher likelihood (26.1%) of FFR-CT ≤0.75 compared to the T2DM group (13.8%). FFR-CT ≤0.75 correlated with CAD-RADS 2.0 (OR = 7.986, 95% CI = 5.466–11.667, cutoff = 3, *p* < 0.001) and noncalcified plaque volume (OR = 1.006, 95% CI = 1.003–1.009, cutoff = 29.65 mm^3^, *p* < 0.001). HRPs were associated with HbA1c levels (OR = 1.631, 95% CI = 1.387–1.918).

**Conclusion:**

AI analysis of CCTA identifies patterns in quantitative plaque characteristics and FFR-CT values. Comorbid HTN exacerbates partially calcified plaques, leading to more severe coronary artery stenosis in patients with T2DM. T2DM is associated with partially noncalcified plaques, whereas HTN is linked to partially calcified plaques.

## Introduction

Type 2 diabetes (T2DM) and hypertension (HTN) both speed up the progression of coronary artery disease (CAD), with patients having either condition showing faster CAD development (Joseph et al., 2017; Leong et al., 2017). Individuals with T2DM are 2 to 4 times more likely to develop HTN, cardiovascular disease, and experience higher mortality rates compared to those without T2DM. These increased risks are observed from the time of T2DM diagnosis and are compared to healthy individuals without T2DM (Lee et al., 2017; Jia and Sowers, 2021). Both T2DM and HTN can compromise endothelial integrity and vascular function, potentially leading to the formation of coronary plaques. This process is driven by increased oxidative stress, endothelial dysfunction, and impaired nitric oxide production, which are well-documented in both conditions (Rochette et al., 2013; Yahagi et al., 2017). Nevertheless, it is crucial to acknowledge that the fundamental mechanisms underlying these two risk factors may exhibit both commonalities and variations. It might be postulated that the attributes of coronary plaques exhibit variability across different exposure factors. Therefore, the quantitative examination of coronary plaques may help identify key biological markers of CAD, such as plaque volume, composition, and the presence of high-risk plaques (HRPs). These markers can serve as indicators of disease severity and potential targets for therapeutic intervention.

Coronary computed tomography angiography (CCTA) is a non-invasive technique used to assess key attributes of coronary plaques, including plaque volume, composition (calcified, non-calcified, or mixed), morphology, and HRPs features. HRPs, which are characterized by thin-capped and lipid-rich content, are associated with acute coronary syndrome (ACS) (Lee et al., 2019; Tomaniak et al., 2020). Identifying HRPs and necrotic cores within coronary plaques exhibiting non-severe stenosis (<50%) is crucial, as recent evidence suggests that a significant proportion of ACS events originate from these plaques, shifting the focus from severe stenosis to early detection of HRPs in less severely narrowed regions to prevent adverse cardiovascular outcomes (Daghash et al., 2020; Virani et al., 2020). The Coronary Artery Disease Reporting and Data System 2.0 (CAD-RADS 2.0) guideline recommends including the HRPs in the diagnostic report to expedite clinical intervention and therapy for HRPs (Cury et al., 2022).

The integration of artificial intelligence (AI) into CCTA has significantly enhanced diagnostic accuracy, particularly in evaluating coronary plaque characteristics and CT-derived fractional flow reserve (FFR-CT). While traditional convolutional neural networks (CNNs) are effective for image classification, they often fall short in providing precise pixel-level predictions. In contrast, the U-Net architecture, with its encoder-decoder structure and skip connections, allows for accurate segmentation, making it especially suitable for the analysis of coronary plaque s and the prediction of FFR-CT on CCTA (Föllmer et al., 2024; Guo et al., 2024). Previous studies (Fairbairn et al., 2018; Mickley et al., 2022; Hashemi et al., 2022) have consistently demonstrated that FFR-CT achieves a diagnostic accuracy of 80–88% and a sensitivity of 81–90%, comparable to invasive FFR. The ADVANCE study showed that patients with moderate stenosis (50–75%) and FFR-CT ≤0.75 frequently require invasive coronary angiography (ICA), while those with stenosis >75% but FFR-CT >0.80 can often avoid it. FFR-CT >0.80 indicates that the stenosis is not functionally significant enough to reduce blood flow and cause ischemia (Fairbairn et al., 2018). Similarly, the FACC study reported that patients with FFR-CT >0.80 had lower rates of revascularization without a significant increase in adverse cardiovascular events (Mickley et al., 2022), underscoring the functional rather than purely anatomical assessment of CAD. Although limited imaging research has specifically examined the impact of HTN on coronary plaque characteristics in patients with T2DM, existing studies have largely relied on manual, qualitative measurements, which are prone to variability and lack precision, particularly due to the absence of FFR-CT integration (Jiang et al., 2022; Tomizawa et al., 2015). This study addresses this gap by utilizing U-Net model to analyze coronary plaque characteristics and FFR-CT on CCTA, providing a comprehensive quantitative assessment of cardiovascular risk in T2DM patients with or without HTN, potentially enhancing risk stratification and management strategies.

## Methods

### Study population

**Figure 1:**
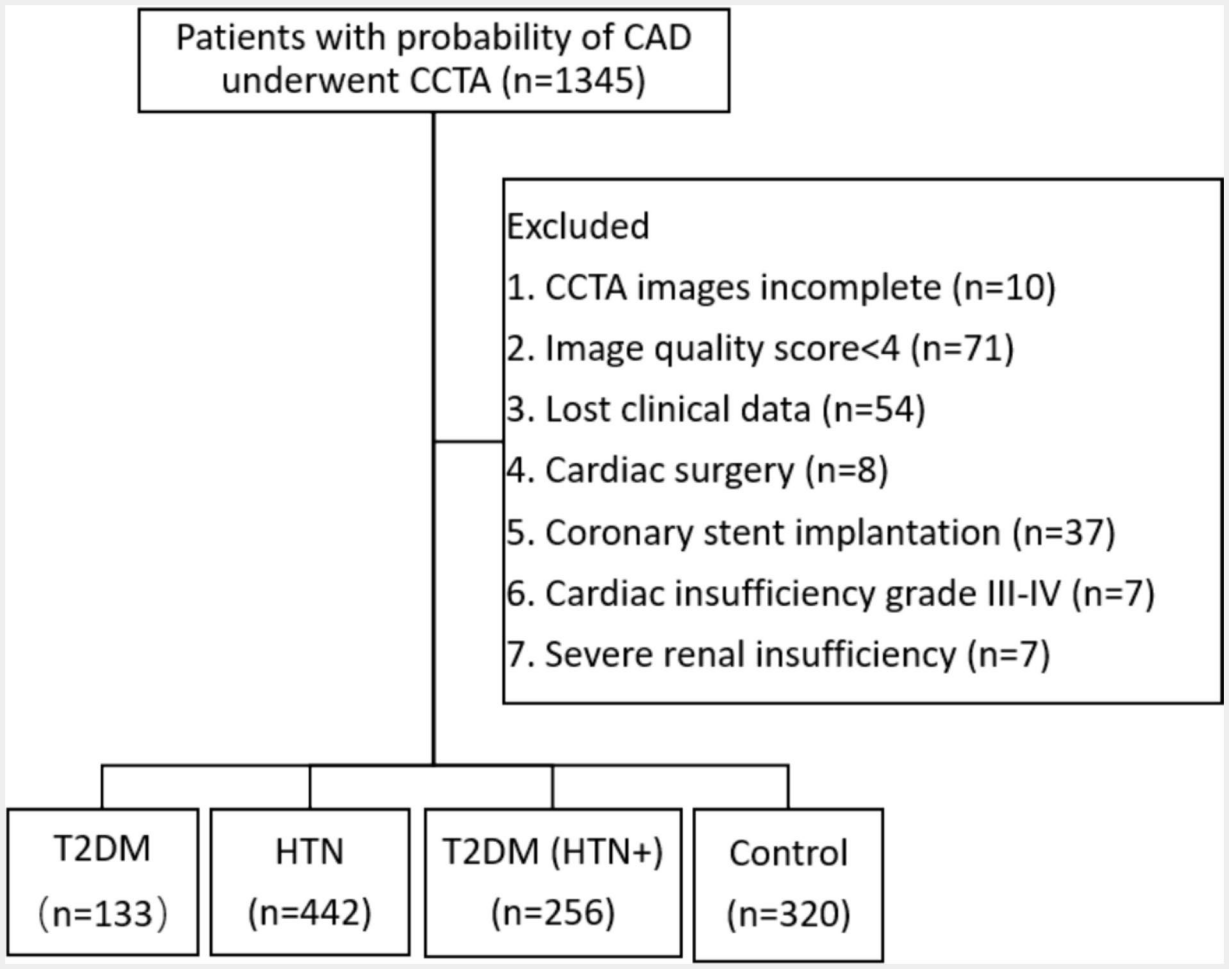
Flowchart of the inclusion and exclusion criteria.

This study was approved by our hospital’s Research Ethics Committee (No. 2023-054-1), and the retrospective study was agreed to exempt patients from signing the informed consent. A cohort of 1,345 patients presenting with clinical suspicion of CAD and undergoing CCTA at our institution between October 2022 and February 2024 was included in this study. The study’s exclusion criteria encompassed several factors: (1) CCTA images incomplete. (2) AI detected the image quality based on a scoring system (1–5), where scores of 4 or 5 were considered suitable for analysis. The exclusion of lower quality images ensures the precision of plaques and FFR-CT analysis, as the algorithm relies on high-quality imaging for accurate measurements. The typical example for the image quality scores was shown in supporting information (Supplementary Figure S1). (3) Absence of clinical data in this study. (4) Prior cardiac surgery involving procedures such as artificial heart valve surgery, coronary artery bypass grafting, and cardiac pacemaker implantation. (5) Previous coronary revascularization and coronary stent implantation. (6) Cardiac insufficiency classified as grade III–IV, because the altered hemodynamics in advanced heart failure could confound the accuracy of FFR-CT analysis. (7) Severe renal insufficiency, because patients received the minimum dose of iodine contrast agent, and the iodine concentration could potentially affect the accuracy of FFR-CT analysis. Finally, 1,151 patients were enrolled in the study (Figure 1), including the T2DM group (*n* = 133), the HTN group (*n* = 442), the T2DM (HTN+) group (*n* = 256), and the control group (*n* = 320). The diagnosis of T2DM was established based on a fasting plasma glucose level of ≥126 mg/dL, a 2-h plasma glucose level of ≥200 mg/dL during an oral glucose tolerance test, or an HbA1c level of ≥6.5%. HTN was diagnosed with a systolic blood pressure of ≥140 mmHg or a diastolic blood pressure of ≥90 mmHg based on at least two separate measurements. The patient’s clinical data was gathered, including their gender, age, dyslipidemia (high levels of total cholesterol, triglycerides, or low-density lipoprotein), smoking, drinking, family history of CAD (first-degree relatives, including parents or siblings), length of illness (determined from the patient’s medical records and self-reported diagnosis by healthcare providers), and body mass index (BMI, kg/m^2^). The following serum biomarkers were measured: low-density lipoprotein cholesterol (LDL-C, mmol/L), hemoglobin A1c (HbA1c, %), and C-reactive protein (CRP, mg/L).

### CCTA scanning protocols

The CCTA examinations were performed by a third-generation dual-source CT scanner (SOMATOM Force, Siemens Healthineers, Germany). The retrospective or prospective coronary artery scanning mode was chosen based on the patient’s respiratory control and heart rate. Automated tube voltage and current modulation (CAREkV, CAREDose4D), collimator 192 × 0.6 mm, field of view 184 × 184 mm, pitch 0.15–0.28 (automatic adjustment based on the heart rate) were utilized. The trigger point was at the center of the descending aorta at a level of 1 cm above the tracheal bifurcation (the trigger threshold was 100 HU), and the acquisition was delayed for 5 s. The RR interval exhibited variability ranging from 30 to 80%. The sector reconstruction time of 66 ms automatically recreated the highest-quality images of the diastolic and systolic periods. The reconstructed slice thickness was 0.75 mm, and the slice increment was 0.5 mm. The convolution used was BV40, and the matrix size was 512 × 512. The contrast agent was administered via a bolus injection through the cubital vein at a rate of 4.0 mL/s. The contrast agent was an iodinated contrast agent (370 mg/mL, BeiLu Pharmaceutical, Beijing, China). The total volume of contrast agent administered varied between 60 and 80 mL. Subsequently, an additional 40 mL of saline solution (75%) was supplied at an equivalent rate.

### CCTA analysis

**Figure 2:**
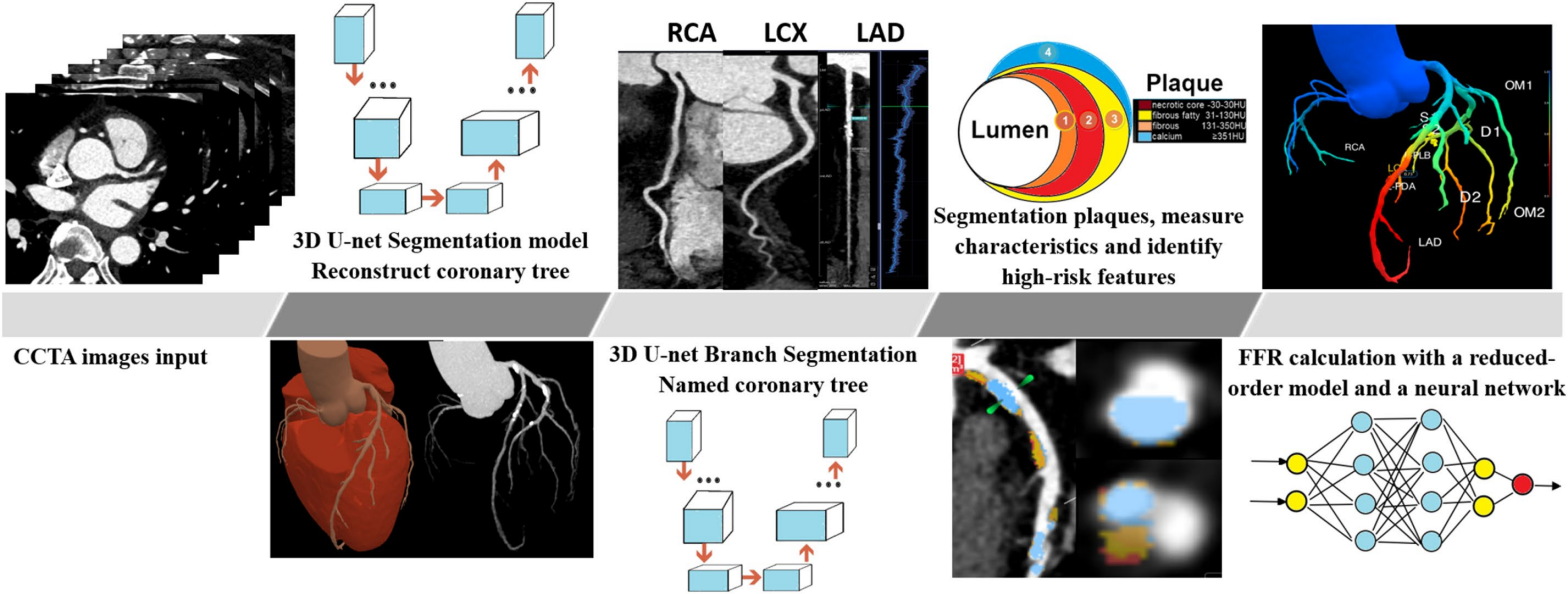
Principle of the fully-automatic plaque characteristics and FFR-CT calculation by AI software.

The superior CCTA images were chosen from the diastolic and systolic periods and subsequently examined using an AI software program (skFFR-CT version v0.6.1, Beijing, China). The process of AI analysis of CCTA is shown in Figure 2. The analysis of FFR-CT from the CCTA includes the following two major steps: (1) automatic coronary artery reconstruction from CCTA images in this work was performed using a modified U-Net. Generally, U-Net is composed of a contracting path and a symmetric expanding path, with skip connections applied between them for feature fusion. The contracting path contains successive down-sampling layers to capture context, while the symmetric expanding path consists of a series of up-sampling layers aimed at recovering localization. In this modified U-Net, bottleneck blocks were inserted into adjacent down-sampling layers of the contracting path, as well as in the symmetric expanding path. Thousands of well-labeled CCTA scans from multiple centers were used to train the modified U-Net model. Additionally, plaque detection and segmentation based on coronary structure, guided by a 3D + 2D convolutional neural network model combined with a fully adaptive receptive field and multi-head self-attention, were performed to accurately segment the coronary vessel lumen. Using this U-Net model, the entire coronary artery tree, including branch vessels with diameters of 1–2 mm, can be automatically and precisely segmented within 2 min. (2) FFR-CT calculation. The distribution of pressure along the centerline of each vessel is calculated in two steps. In the first step, the pressure is calculated with a reduced-order model (Guo et al., 2024). In the second step, the calculated pressure is further processed with a neural network, which is trained to minimize the discrepancy of pressure between the reduced-order model results and 3D computational fluid dynamics (CFD) or invasive measurement results. The major steps of the FFR-CT computation were shown in the supporting information (Supplementary Figure S2). FFR-CT ≤0.75 was defined as myocardial-specific ischemia (Fairbairn et al., 2018). Images with an Agatston calcification score greater than 1,000 were excluded from FFR-CT computation, but plaque calculation was still conducted.

Plaque characteristics, including total plaque length, total plaque volume, plaque component volume, and proportion, were calculated by AI software (Figure 2; Supplementary Figure S3). The components of the plaque were separated based on their Hounsfield unit (HU) values: calcium volume (CV) ≥351 HU, fibrous volume (FV) between 131 to 350 HU, fibrous fatty volume (FFV) between 31 to 130 HU, and necrotic core volume (NCV) between −30 to 30 HU (de Graaf et al., 2013). The HRPs are defined as plaques that meet at least the following two conditions: low-density plaque (NCV >1 mm^3^ or >40% of the total plaque volume), positive remodeling (remodeling index ≥1.1), punctate calcification (<3 mm), and napkin ring sign (high-density ring sign formed around low-density plaque) (Yang et al., 2022). Coronary stenosis severity classification was assessed according to the CAD-RADS 2.0 (0–5, 0 = 0%, 1 = 1–24%, 2 = 25–49%, 3 = 50–69%, 4 = 70–99%, and 5 = 100%). The calculation of the segment involve score (SIS) and segment stenosis score (SSS) was performed. The SIS ranges from 0 to 16, while the SSS ranges from 0 to 48, which is obtained by summing the product of SSS and coronary stenosis severity (1 < 50, 50% ≤ 2 < 70%, 3 ≥ 70%). The determination of HRPs was collaboratively conducted by two radiologists who were ignorant of the clinical data (with 8 and 20 years of experience, respectively). Consultation determined the ultimate result in situations when the two radiologists encountered disagreement.

### Statistical analysis

The data was analyzed statistically using IBM SPSS Statistics 27 and GraphPad Prism 10 software. The continuous data was displayed as the mean ± standard deviation (SD) or median and interquartile range (IQR), depending on whether it matched a normal distribution. The normality of the data was assessed using the Kolmogorov–Smirnov test. The categorical data were presented as absolute frequencies and proportions. The one-way ANOVA was implemented to compare continuous variables across distinct groups, assuming the data adheres to a normal distribution. A post-hoc test (Tukey’s HSD or Dunn–Bonferroni) was conducted following the one-way ANOVA to identify which specific groups differed from each other. The nonparametric Kruskal–Wallis *H* test was utilized for statistical analysis in scenarios where the data does not conform to a normal distribution. Logistic regression analysis investigates the risk factors associated with FFR-CT ≤0.75 and HRPs. *p*-values<0.05 are considered statistically significant.

## Results

### Baseline characteristics

Table 1 summarizes the baseline characteristics of the study participants. This study encompassed an overall population of 1,151 patients. There were significant discrepancies in age (*p* < 0.001), but not in gender, BMI, dyslipidemia, smoking, alcohol consumption, or family history of CAD among the four groups (*p* > 0.05). A statistically significant disparity in disease duration was seen between groups (*p* = 0.002). There was no statistically significant difference in the location of the narrowest coronary artery among the four groups. With a statistically significant difference (*p* < 0.001), the SIS and SSS showed the highest values in the T2DM (HTN+) group, then the T2DM and HTN groups, and the lowest in the control group.

**Table 1:** Baseline characteristics.

	T2DM (*n* = 133)	HTN (*n* = 442)	T2DM (HTN+) (*n* = 256)	Control (*n* = 320)	*p*
Age (years)	66.01 ± 10.39	67.30 ± 8.79	67.14 ± 9.26	62.22 ± 11.02	<0.001^a^
Male, *n* (%)	62 (46.6)	180 (40.7)	119 (46.5)	144 (45.0)	0.383^b^
BMI, kg/m^2^	22.67 ± 2.54	22.46 ± 1.85	22.82 ± 2.09	22.53 ± 2.05	0.135^a^
Duration (years)	9.28 ± 7.78	11.31 ± 10.13	8.45 ± 7.43	0	<0.001^a^
Dyslipidemia, no (%)	37 (27.8)	114 (32.6)	75 (29.3)	77 (24.1)	0.527^b^
Current smoker, *n* (%)	29 (21.8)	78 (17.6)	48 (18.8)	40 (12.5)	0.058^b^
Current drinker, *n* (%)	12 (9.0)	40 (9.0)	22 (8.6)	20 (6.3)	0.529^b^
Family history, *n* (%)	13 (9.8)	22 (5.0)	18 (7.0)	16 (5.0)	0.156^b^
Narrowest artery, *n*					
LM	0	9	3	6	0.091^b^
LAD	70	225	167	91	
RCA	16	39	35	12	
LCX	10	17	17	7	
RI	0	1	1	0	
D (1–2)	0	4	3	2	
OM (1–2)	0	1	3	0	
SIS^c^	2 (4)	1 (3)	4 (5)	0 (1)	<0.001^b^
SSS^c^	3 (7)	1 (3)	6 (12)	0 (1)	<0.001^b^

a One-way ANOVA for analysis.

b Non parametric tests for analysis.

c Median (IQR).

### Plaque characteristics

**Figure 3:**
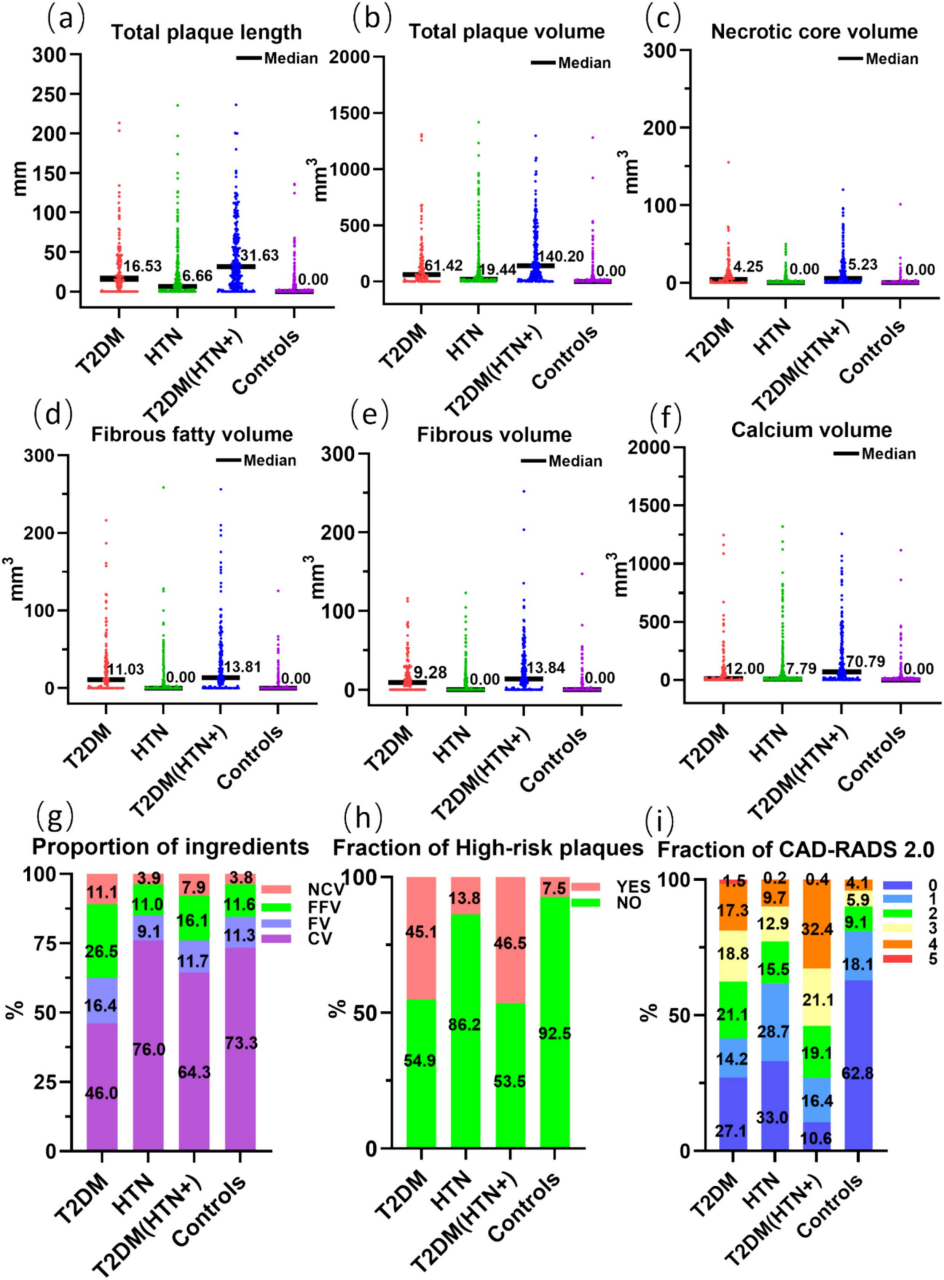
Comparison of the tot al length (a) and volume (b) of coronary artery plaques, the volume (c–f) and proportion (g) of plaque components, the fraction of high-risk plaques (h), and CAD-RADS 2.0 (i) across four groups. NCV, necrotic core volume; FFV, fibrous fatty volume; FV, fibrous volume; CV, calcium volume.

**Figure 4:**
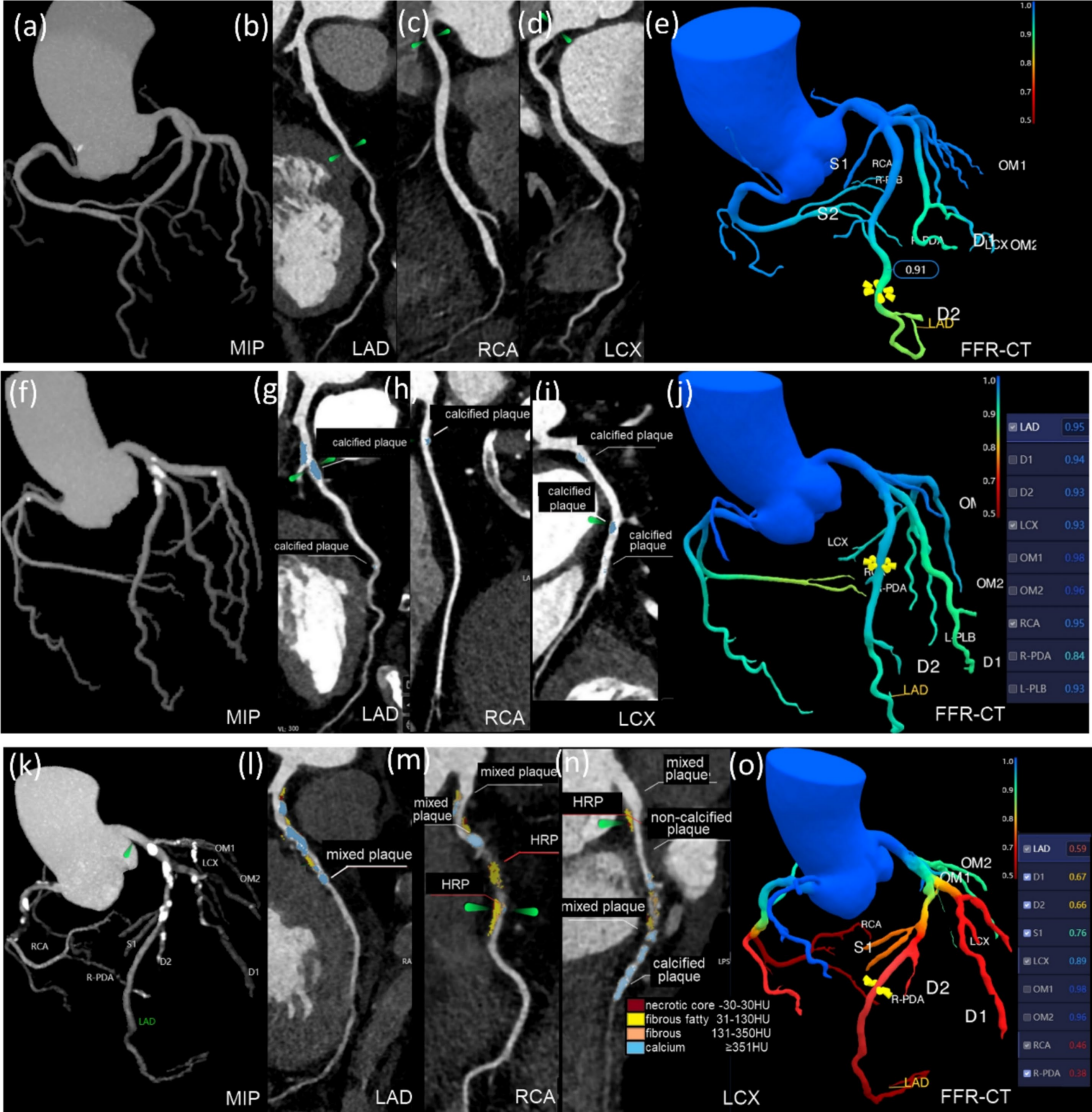
Groups differed in plaque characteristics and FFR-CT on CCTA images. (a–e) This 46-year-old male belongs to the control group. The LAD, RCA, and LCX have no restriction on the CCTA, and the FFR-CT >0.75. (f–j) This 74-year-old female had hypertension for 3 years. CCTA reveals calcified plaques on LAD, RCA, LCX, and OM1. Plaques are 168.8 mm^3^ in volume and 26.23 mm in length. The proximal LAD exhibited moderate constriction, with CAD-RADS 2.0 classification 3, SSS 5, SIS 6, and the FFR-CT >0.75. (k–o) This 74-year-old male had T2DM for 20 years. CCTA scans show calcified, mixed, and HRPs in the LAD, RCA, LCX, D1-2, and OM1. The plaque is 556.42 mm^3^ in volume and 94.64 mm in length. The core necrotic volume is 26.67 mm^3^, the fiber lipid content is 121.22 mm^3^, the fiber volume is 79.47 mm^3^, and the calcification volume is 329.06 mm^3^. The middle RCA, proximal LAD, and middle and distal LCX have severe stenosis, with CAD-RADS 2.0 classification 4, SIS 8, and SSS 18. FFR-CT ≤0.75 was seen in the RCA, LAD, D1, and D2.

The quantitative characteristics of coronary plaques across groups are illustrated in Figure 3. The T2DM group exhibited a higher percentage of noncalcified plaque volume (54.0%) compared to calcified plaque (46.0%), suggesting a higher vulnerability to rupture. In contrast, the HTN group showed a predominance of calcified plaques (24.0% noncalcified vs. 76.0% calcified), indicating greater plaque stability but potential procedural challenges. The T2DM (HTN+) group had a mixed plaque composition (35.7% noncalcified vs. 64.3% calcified). When combined with HTN, coronary plaques in T2DM patients predominantly presented as an increase in calcified plaques, with values of 12.00 mm^3^ and 70.79 mm^3^, respectively. The detailed comparative analysis between groups is presented in Supplementary Tables S1, S2. The T2DM (HTN+) group exhibited an average NCV of 5.23 mm^3^ versus 4.25 mm^3^ for the T2DM group, which was not statistically significant (*p* > 0.05) (Supplementary Table S3). The probability of HRPs in the T2DM and T2DM (HTN+) groups is higher compared to the HTN and control groups, with no significant difference between the T2DM and T2DM (HTN+) groups (*p* > 0.05). T2DM (HTN+) exhibited the highest CAD-RADS 2.0 classification scores. Figure 4 illustrates the plaque characteristics and FFR-CT fluctuations on CCTA images among groups.

### Risk factors associated with FFR-CT ≤0.75

**Figure 5:**
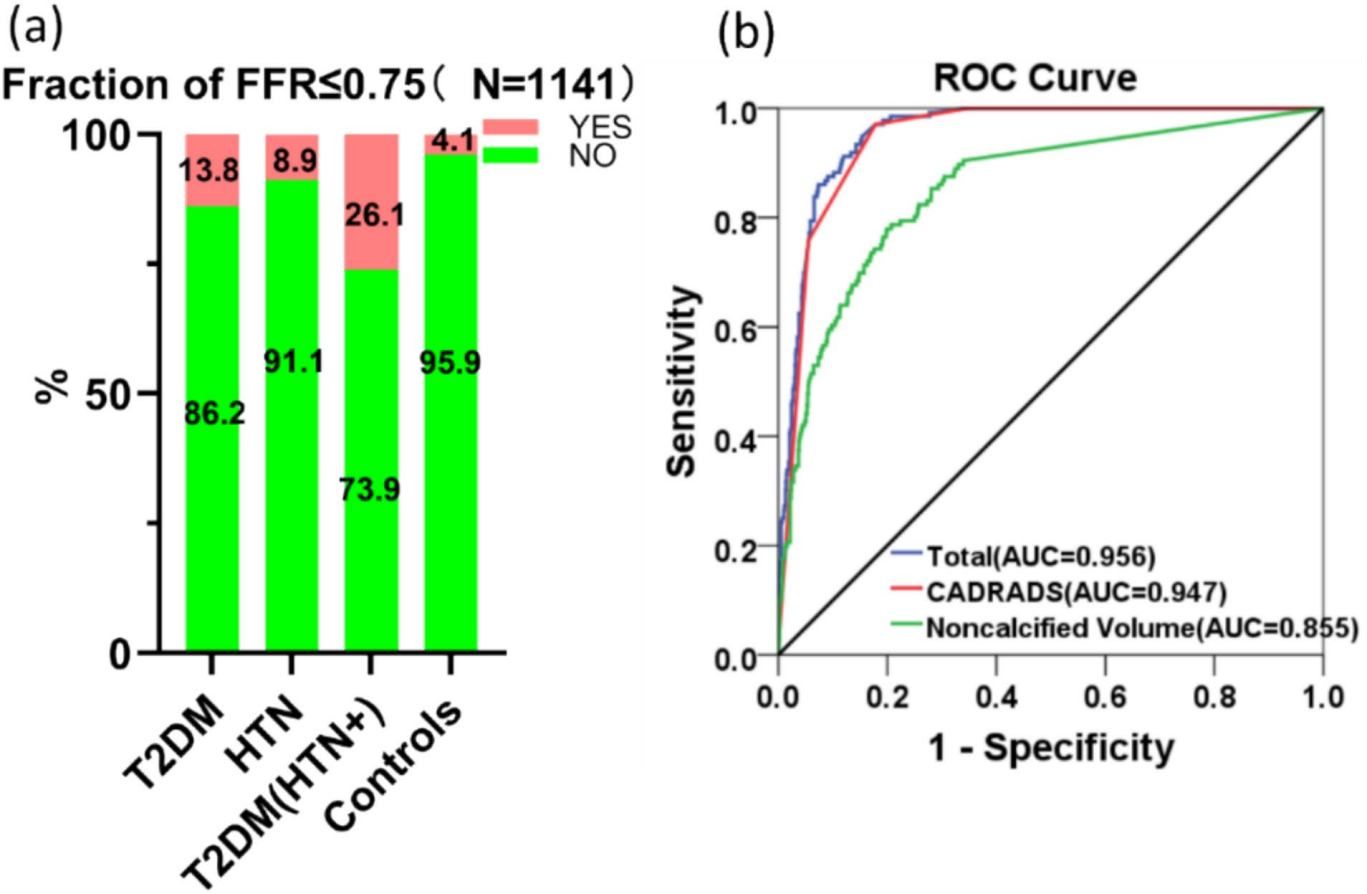
The comparison between groups (a) and the AUC curve (b) generated by logistic regression analysis of risk factors for FFR-CT ≤0.75 are displayed.

In the AI analysis procedure, 10 cases were removed from the FFR-CT calculation because their Agatston calcification score surpassed 1,000. When comparing the control group (13/319, 4.1%), the T2DM group (18/130, 13.8%), and the HTN group (39/439, 8.9%), the T2DM (THN+) group exhibited the highest odds (66/253, 26.1%) of having an FFR-CT ≤0.75 (Figure 5a), indicating more severe functional impairment in coronary circulation due to the combined effect of T2DM and HTN. Nevertheless, among the three remaining groups, no statistical significance was identified. Pair-wise correlations between variables were checked using the variance inflation factor (VIF), ensuring all included variables had a VIF <5, indicating no significant multicollinearity. An analysis of 17 risk factors for FFR-CT ≤0.75 incorporated 1,141 instances into a logistic regression model. Table 2 demonstrated a statistically significant positive association between FFR-CT ≤0.75 and CAD-RADS 2.0 [Regression coefficient *B* (*B*) =2.078, adjusted odds ratio (OR) =7.986, 95% CI = 5.466–11.667, cutoff value = 3, *p* < 0.001] and a relatively weak positive correlation with noncalcified plaque volume (*B* = 0.006, adjusted OR = 1.006, 95% CI = 1.003–1.009, cutoff value = 29.65 mm^3^, *p* < 0.001). The AUC value for the logistic regression model is 0.956 (Figure 5b). The formula used in the model is as follows: ln (*p*/1 − *p*) = −8.221 + 2.078 * CADRADS+0.006 * NCV. In this equation, *p* represents the probability of FFR-CT ≤0.75.

**Table 2:** Risk factors analysis utilizing univariate and multivariate logistic regression for FFR-CT ≤0.75 (*N* = 1,141).

Variates	Univariate analysis			Multivariate analysis (forward: LR)		
	OR	95% CI	*p*-value	OR	95% CI	*p*-value
Gender (male = 1)	0.487	0.339–0.702	<0.001			0.162
Age	1.038	1.018–1.059	0.001			0.079
BMI	1.006	0.922–1.098	0.892			0.597
CADRADS (per + 1)	9.127	6.320–13.183	<0.001	7.986	5.466–11.667	<0.001
HRPs	0.114	0.077–0.168	<0.001			0.886
Total plaque length	1.041	1.034–1.047	<0.001			0.664
Total plaque volume	1.006	1.005–1.007	<0.001			0.496
Noncalcified volume^a^	1.018	1.015–1.021	<0.001	1.006	1.003–1.009	<0.001
Calcified volume	1.006	1.005–1.007	<0.001			0.496
T2DM	3.814	2.631–5.529	<0.001			0.164
HTN	2.412	1.585–3.670	<0.001			0.683
Dyslipidemia	1.561	1.067–2.285	0.022			0.674
Current smoker	3.132	2.110–4.648	<0.001			0.957
Current drinker	2.805	1.682–4.677	<0.001			0.615
Family history of CAD	1.273	0.635–2.551	0.497			0.853
SIS	1.614	1.502–1.735	<0.001			0.876
SSS	1.330	1.281–1.381	<0.001			0.256

a Noncalcified volume = Fibrous volume + Fibrous fatty volume + Necrotic core volume.

### Correlation between HRPs and serum biomarkers

**Figure 6:**
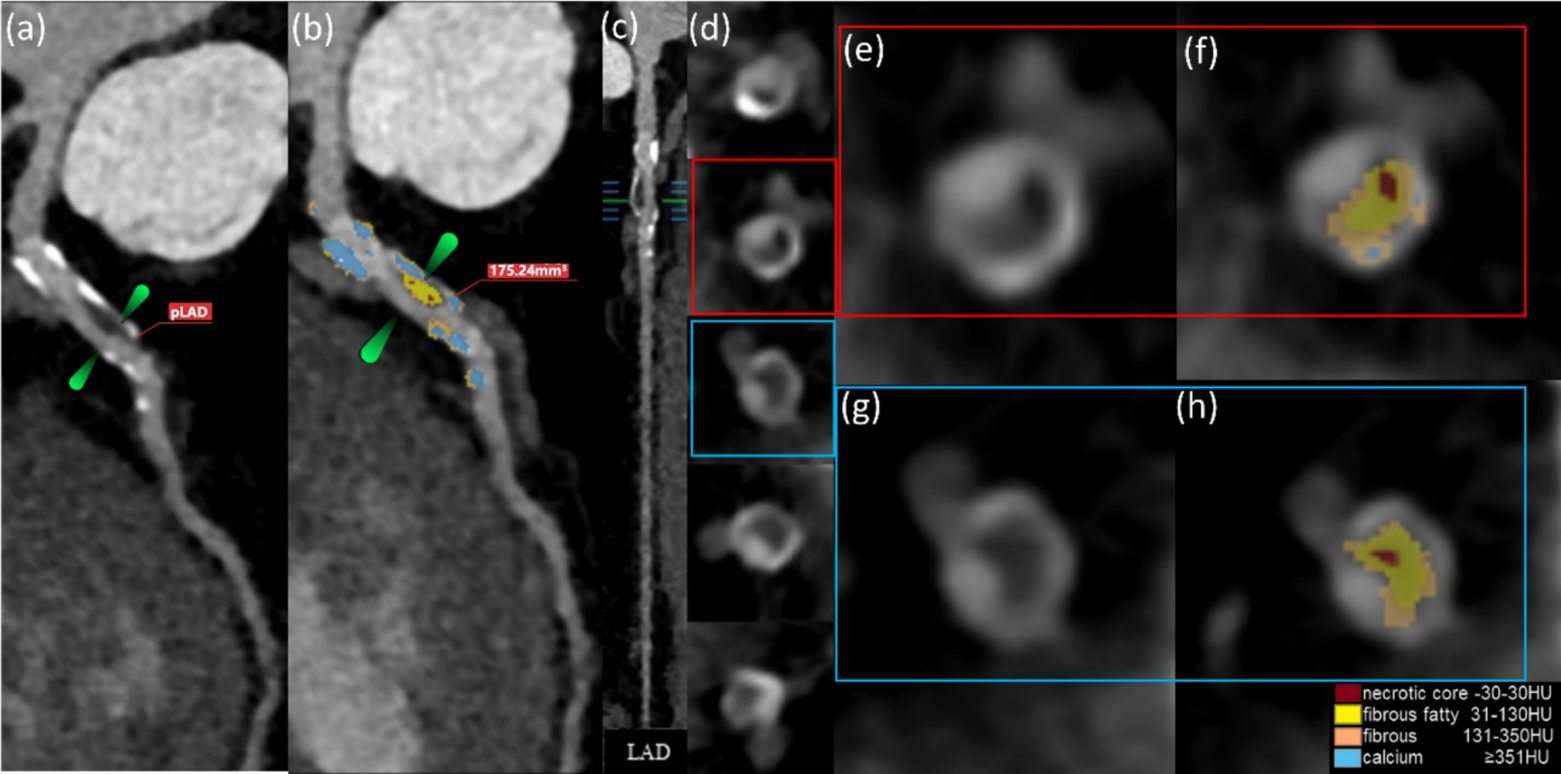
The AI computations quantitatively represent the HRPs on CCTA images (a–h). These CCTA images were obtained from a 50-year-old male with T2DM for 5 years and (a) HbA1c level of 7.0%. The volume of proximal LAD plaques is 175.24 mm^3^ (b). Plaque component classification by AI is contingent upon HU.

Figure 6 shows that the U-Net model can accurately separate the compounds inside HRPs by using HU threshold segmentation. From the total cohort of 1,151 patients, a sample of 439 was selected, as only these individuals had all three blood biomarkers (HbA1c, LDL-C, and CRP) measured. Logistic regression was performed to assess the relationship between these biomarkers and HRPs. Of the 439 patients, 188 (42.8%) exhibited HRPs on CCTA images. The findings demonstrated a favorable correlation between HRPs and HbA1c (*B* = 0.489, OR = 1.631, 95% CI: 1.387–1.918, cutoff value = 6.9%, *p* < 0.001). However, no correlation was observed between HRPs and CRP or LDL-C (Table 3).

**Table 3:** Logistic regression (enter) correlation between three blood biomarkers and HRPs.

Variates	Mean ± SD	OR	95% CI	*p*-value
HbA1c, %	6.97 ± 1.74	1.631	1.387–1.918	<0.001
LDL-C, mmol/L	2.74 ± 0.96	1.000	0.811–1.234	1.000
CRP, mg/L	3.73 ± 7.54	1.005	0.977–1.034	0.717

## Discussion

The U-Net model enables rapid, multiparametric analysis of CCTA images within minutes, providing diagnostic information and supporting large-scale epidemiological studies. Traditional methods provide basic plaque morphology and stenosis grading. AI adds detailed plaque component quantification and FFR-CT analysis, improving risk stratification and enabling more personalized clinical decisions. This research focuses on integrating U-Net for comprehensive plaques and FFR-CT analysis in T2DM patients, with an emphasis on the role of hypertension. Our findings suggest that T2DM (HTN+) increased the volume of calcified plaques but not noncalcified and necrotic core plaques than T2DM patients. T2DM patients exhibited a higher risk of HRPs compared to HTN and control groups. The CAD-RADS, SIS, SSS, and the probability of FFR-CT ≤0.75 in T2DM (HTN+) were significantly higher than in T2DM, indicating a concomitant development of coronary atherosclerosis.

In this work, it was observed that T2DM (HTN+) results in an augmentation in calcified plaque volume and more serious coronary atherosclerosis than T2DM. This is consistent with previous research results (Jiang et al., 2022). Complex hemodynamic feedback in T2DM (HTN+) individuals worsens cardiovascular disease (Ferrannini and Cushman, 2012; Climie et al., 2019). The probability of FFR-CT ≤0.75 in T2DM (HTN+) was found to be higher than in T2DM or HTN alone, which is a novel finding. The additional FFR-CT examination may increase the diagnosis cost of CAD patients (Curzen et al., 2021; Hlatky et al., 2023), and whether an FFR-CT examination is necessary for patients should be discussed. This study suggests that the need for FFR-CT evaluation is primarily driven by CCTA imaging findings, rather than the patient’s clinical history (Table 2), especially when the CAD-RADS 2.0 score is ≥3. FFR-CT ≤0.75 indicates a higher likelihood of requiring intervention, while FFR-CT >0.80 may avoid unnecessary invasive procedures (Fairbairn et al., 2018; Mickley et al., 2022). Additionally, U-Net algorithms enhance the accuracy and consistency of analyzing large-scale CCTA datasets. Unbiased results are ensured by the algorithm’s reliance on objective image analysis rather than subjective interpretation by radiologists.

Previous studies have shown AI’s effectiveness in improving FFR-CT diagnostic accuracy for CAD (Guo et al., 2024) and identifying HRPs (Föllmer et al., 2024). This study uniquely integrates the analysis of noncalcified and calcified plaques, HRPs, and FFR-CT in T2DM patients with and without HTN, uncovering compounded risk factor patterns. Notably, the study highlights the association between FFR-CT ≤0.75 and increased noncalcified plaque volume, suggesting a higher risk of coronary ischemia even in the absence of significant calcification. Quantitative characteristics of coronary plaques using CCTA can facilitate personalized strategies for prevention, therapy, and early drug intervention in patients with or without T2DM. Our findings indicated that patients diagnosed with T2DM exhibited an elevated susceptibility to the formation of noncalcified plaques compared to those with HTN. Although this study observed a greater percentage of calcified plaques in individuals with HTN, HTN still represents a risk factor for the formation of noncalcified plaques. Miller et al. (2023) found that both T2DM and HTN were associated with the formation of noncalcified plaques, with an OR of 2.192 and 1.613, respectively, which indicated a heightened risk of developing noncalcified plaques in individuals with T2DM. Pathological studies have revealed that the coronary arteries of people with T2DM have a larger necrotic core and more obvious vascular inflammation, mostly made up of macrophages and T lymphocytes (Yahagi et al., 2017). Even without hyperlipidemia, diabetes stimulates macrophages to transform into inflammatory cells and increase the lipid core (Tabas, 2010). Additionally, hyperglycemia may increase vascular smooth muscle cell (VSMC) proliferation, migration, and reactivity, promoting atherosclerosis (Climie et al., 2019). HTN activates L-type calcium channels in VSMCs, which raises intracellular calcium. Excessive intracellular calcium quickly decomposes VSMCs’ mitochondria and structural components, resulting in calcium accumulation in elastic fibers (Miller et al., 2023; Tabas, 2010; Godfraind, 2017; Tedla et al., 2017; Zhou et al., 2018; Zhu et al., 2018). Plaque treatment improves cardiovascular outcomes in patients with T2DM. Statins inhibited noncalcified plaque growth, converted the HRPs phenotype to calcified plaques, and attenuated coronary atherosclerosis (Ferrannini and Cushman, 2012; Lee et al., 2018; van Rosendael et al., 2021).

Enhancing plaque treatment by targeting the amount of noncalcified plaque volume can effectively improve coronary atherosclerosis (Kini et al., 2013). Previous studies (Okada et al., 2015; Li et al., 2020) indicated that the instability and inconsistency of glucose and HbA1c in individuals with T2DM might heighten the susceptibility to coronary culprit plaque, or HRPs. In this work, we found that individuals with HbA1c ≥6.9% are more likely to possess HRPs, which supports the role of glycemic metabolism abnormalities as a strong independent risk factor for HRPs and is consistent with previous studies (Conte et al., 2021; Heinsen et al., 2021). It was observed that there was a favorable association between FFR-CT ≤0.75 and noncalcified plaque volume (Table 2). When assessing the impact of coronary artery stenosis on myocardial ischemia using CCTA, it is important to take the volume of the noncalcified plaque volume into account, especially for coronary atherosclerosis patients with an Agatston calcium score of 0. Gaur et al. (2016) found that FFR ≤0.80 was associated with both coronary stenosis severity and noncalcified plaque volume, indicating a risk factor for myocardial ischemia. Lu et al. (2023) demonstrated that CCTA could estimate plaque NCV to identify residual risk in non-ST-elevation myocardial infarction patients.

The quantification of CCTA using the U-Net model is non-invasive and more cost-effective, as it reduces the need for unnecessary invasive intravascular procedures (Curzen et al., 2021). While invasive coronary angiography cannot assess plaque composition and some plaques that do not restrict blood flow can still cause adverse events (Erlinge et al., 2021), intravascular ultrasonography and optical coherence tomography can identify plaques but are invasive with a 0.4–1.6% complication rate (Waksman et al., 2019). This model combines U-Net driven CAD-specific functional parameters with quantitative CCTA assessment, offering new diagnostic insights for coronary heart disease. This approach has significant potential for predicting coronary artery disease and improving patient outcomes (Guo et al., 2023). The U-Net model has the capability to aggregate data from multiple patients, enabling the identification of trends and patterns in plaque characteristics and FFR-CT values. Although this analysis is based on a large single-center cohort, AI analysis of CCTA allows for a more comprehensive identification of trends in plaque characteristics and FFR-CT values. This enables earlier, more personalized interventions, tailored medication regimens, and more precise decision-making regarding invasive procedures.

### Limitations of the study

There are some limitations to this study. It is important to note that there may be variations or discrepancies in the findings of a single-center study. Therefore, it is necessary to conduct additional research involving multiple centers to obtain more comprehensive and reliable results. On the other hand, it should be noted that non-invasive FFR-CT was not validated using invasive FFR. The AI software used in this study has been licensed by the National Medical Products Administration (NMPA) in China and its accuracy was validated during the initial software development phase. Furthermore, the comparison between the measurement of coronary plaque and invasive intracoronary plaque imaging has yet to be conducted. Nevertheless, it is important to note that in the present investigation, stringent measures were taken to ensure the image quality of the included CCTA scans (4–5 points). Additionally, the research team acknowledged the accuracy of coronary plaque delineation achieved in this study. Finally, coronary plaques and FFR-CT were discussed in this investigation, but the predictive risk factors for major adverse cardiovascular events (MACE) in T2DM patients with or without HTN are not addressed. We will continue to monitor patient data, with MACE as the endpoint event.

## Conclusion

Our research indicates that patients with diverse risk factors exhibit variations in coronary plaques and hemodynamics on CCTA. Utilizing U-Net algorithms, AI significantly enhances CCTA by accurately quantifying coronary plaques, including non-calcified plaques with an Agatston score of 0. In patients with T2DM, HTN confers an enhanced risk of coronary atherosclerosis. Coronary calcified plaques, stenosis severity, and FFR ≤0.75 deteriorate in T2DM (THN+) patients. T2DM patients exhibited a larger volume of necrotic core and an elevated risk of developing HRPs than HTN. HTN patients had a higher prevalence of partly calcified plaques. CAD-RADS 2.0 ≥3 is recommended to perform FFR-CT evaluations. AI technology analysis of CCTA has broad application prospects in improving the diagnosis and management of cardiovascular diseases.

## Data Availability

The original contributions presented in the study are included in the article/Supplementary material, further inquiries can be directed to the corresponding authors.
