# Supplementary material for: Impact of hypertension on coronary artery plaques and FFR-CT in type 2 diabetes mellitus patients: evaluation utilizing artificial intelligence processed coronary computed tomography angiography
Source: Front Artif Intell. 2024 Oct 23;7:1446640. doi: 10.3389/frai.2024.1446640 (PMC11537896; doi:10.3389/frai.2024.1446640)
Supplement: Supplementary file 3 [file Data_Sheet_1.docx]

Supporting Information

**METHODS**


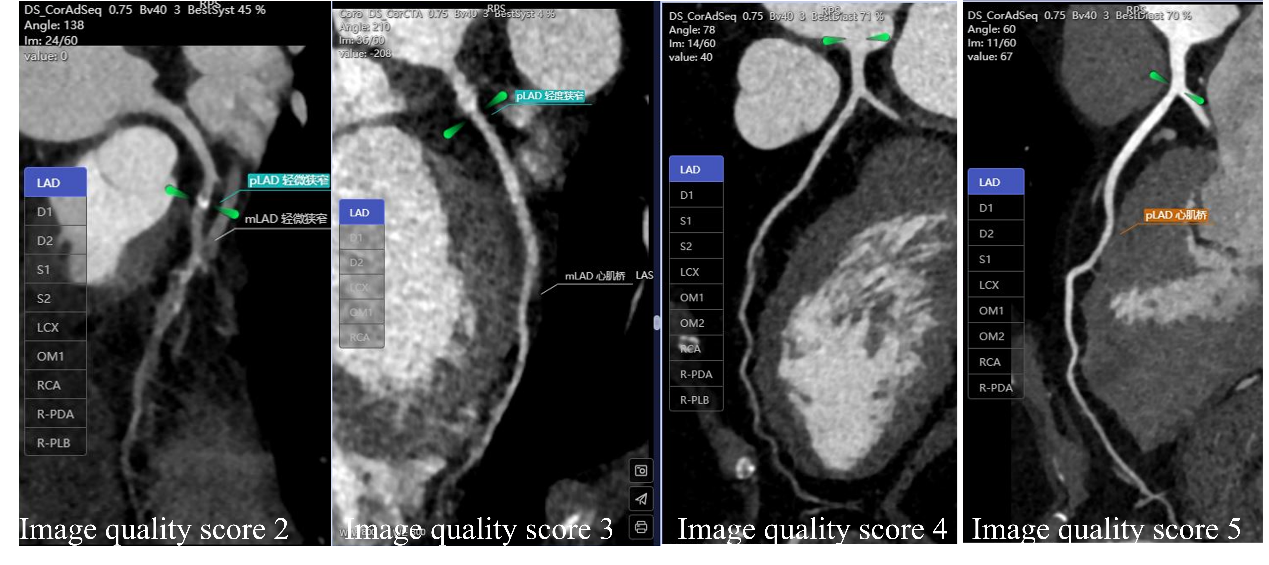


Fig. S1 The typical example for the image quality scores

CCTA analysis

1. Principle of the fully-automatic FFR-CT calculation

The FFR-CT calculations were based on a research version of a dedicated software (skFFR-CT version v0.6.1, Beijing, China). The analysis of FFR-CT from CCTA includes the following two major steps.

1) Automatic coronary artery reconstruction (coronary image post-processing).

The coronary artery reconstruction from CCTA images in this work was performed by a modified U-net. Generally, U-net is composed of a contracting path and a symmetric expanding path, between which there are skip connections applied for feature fusion. The contracting path contains successive down-sampling layers used to capture context and the symmetric expanding path which consists of a series of up-sampling layers aiming to recover localization. In this modified U-net, bottle-neck blocks are inserted into adjacent down-sampling layers of the contracting path, and so do in the symmetric expanding path. Then, 2144 well-labeled CCTA scans from muti center are applied for training our modified U-net model. At the same time, the plaque detection and segmentation based on a coronary structure guided by 3D + 2D convolutional neural network model combining with fully adaptive receptive field and multi-head self-attention were performed to get an accurate coronary vessel lumen. Based on this U-net, the precise segmentation of whole coronary artery tree including branch vessels 1-2 mm in diameter can be reconstructed automatically within 2 minutes.

2) FFR-CT calculation.

Using the segmentation information obtained from the reconstruction procedure and personalized physiological parameters, a reduced-order FFR-CT calculation method modified by the machine learning prediction is adopted to calculate FFR. The distribution of pressure along the centerline of each vessel is calculated in two steps (Fig. S2). In the first step, the pressure is calculated with a reduced-order model ^[13]^. It was modified by considering the effect of heart rate. The whole coronary is divided into stenotic and non-stenotic regions, and the pressure-drop of each region is computed with the regional lumen segmentation features. The inlet pressure is set to the patient-specific mean artery pressure or 100 mmHg if it is not available. Pateint-specific flow resistances are prescribed to all outlets. The value of flow resistance at resting condition is derived from the flow rate at each outlet and mean artery pressure. Total coronary flow rate is assumed to be determined with left ventricle mass, and is distributed to each outlet following Murray’s Law. The hyperemic condition is simulated by appropriately reducing the flow resistance. In the second step, the calculated pressure is further proceeded with a neural network which is trained to minimize the discrepancy of pressure between the reduced-order model results and 3D computational fluid dynamics (CFD) or invasive measurement results. The calculated pressure and coronary segmentation features are input to the neural network, and the updated distribution of pressure are output. It is important to note that the segmentation features used in these two steps are not the same. In fact, the second step which introduced a well-designed neural network can make use of more segmentation features that have not been used in the first step, thus it can further improve the accuracy of the reduced-order model.


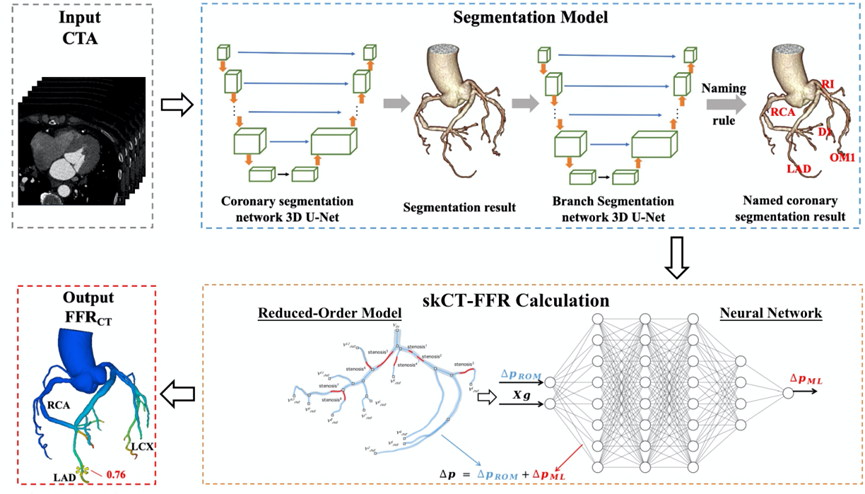


Fig. S2 The pipeline of fully-automatic FFR-CT calculation. CCTA images are input into a segmentation model to reconstruct the coronary tree, and then the named coronary tree segmentation features are extracted to calculate the FFR values. The pressure *d*_rop_ ($\Delta p$) caused by blood flow through the non-stenotic and stenotic regions is calculated by summing up the pressure drop derived with a reduced order model (${\Delta p}_{ROM}$) and the pressure drop inferred with a neural network (${\Delta p}_{ML}$). ${\Delta p}_{ROM}$ is derived with segmentation features and patient-specific physiological information. ${\Delta p}_{ML}$ is inferred with the ${\Delta p}_{ROM}$ and segmentation features by the neural network (Provided by skFFR-CT company).

2. Novelty of the fully-automatic FFR-CT

This thoroughly automatic pipeline to calculate FFR-CT is developed by combining the precise and rapid coronary tree reconstruction and FFR-CT calculation processes. No user intervention means that the results are consistent without subjective bias. These advantages are achieved mainly by two innovative technologies. The first one is a fully automated coronary tree reconstruction based on a modified 3D deep convolutional neural network. The second technique is a novel FFR-CT alogrithm that incorporates a reduced-order model and a fully connected neural network, which allows a significant reduction in computational time compared to other 3D fluid dynamics calculations and improves the overall performance.

3. An example of using AI to analyze CCTA.

FFR-CT, Plaque characteristics, including total plaque length, total plaque volume, plaque component volume, and proportion, were calculated by AI software (Fig. S3).


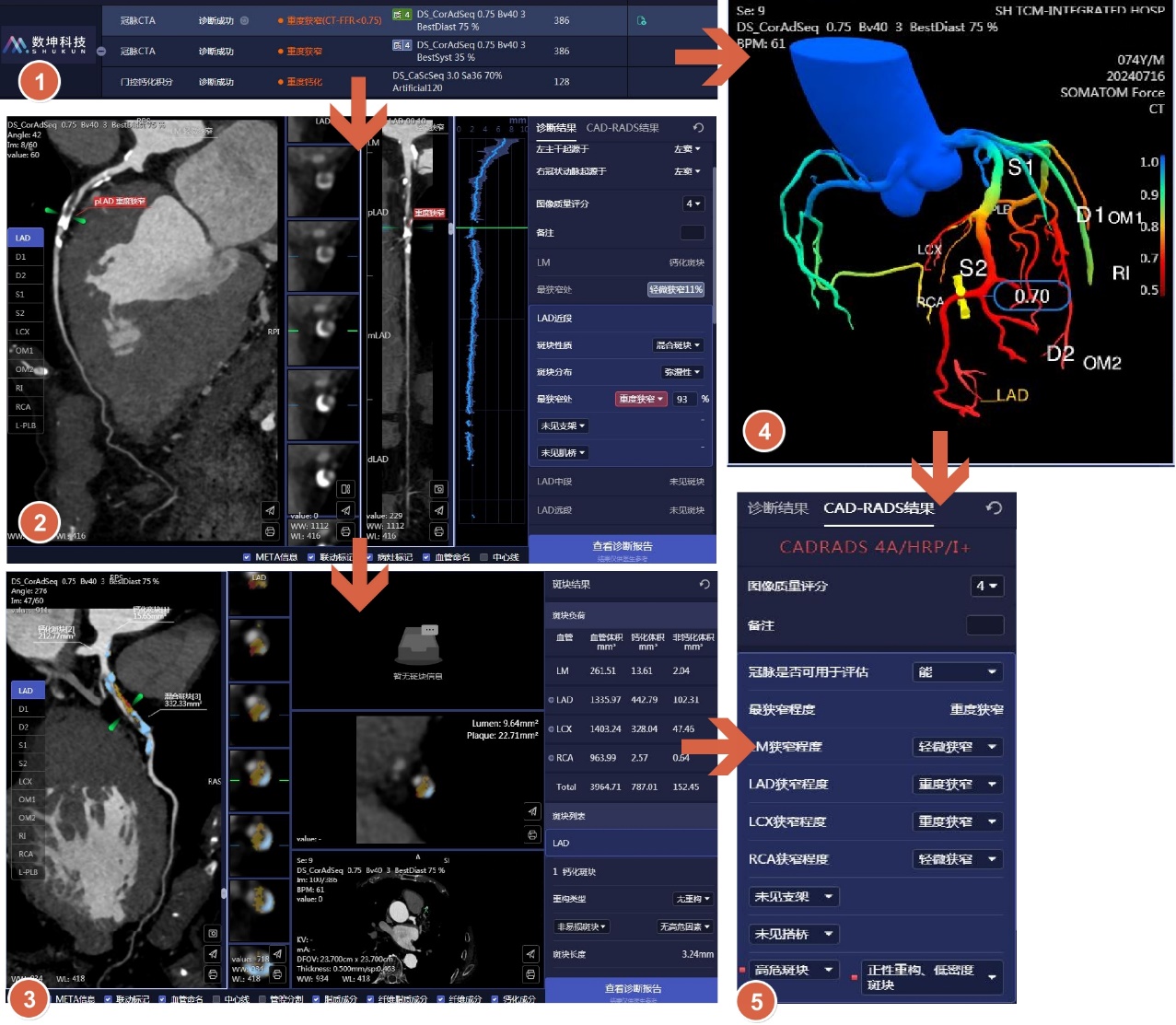


Fig. S3 demonstrates how AI software can comprehensively evaluate CCTA images and obtain multiparametric information: 1. Input the patient data into the AI system, and calculations are completed within 2 minutes. 2. Results of coronary segmentation and stenosis severity are displayed. 3. Quantitative assessment of plaques, including calcified and non-calcified plaques across the entire coronary tree, as well as the identification and evaluation of vulnerable plaques. 4. Calculation of FFR-CT. 5. The final output is provided according to CAD-RADS 2.0.

Reference

[13] Guo B, Jiang M, Guo X, et al. Diagnostic and prognostic performance of artificial intelligence-based fully-automated on-site CT-FFR in patients with CAD. Sci Bull (Beijing). 2024 May 30;69(10):1472-1485. doi: 10.1016/j.scib.2024.03.053.

| **4. Table S1**  **Table S1 Descriptives of plaque characteristics between groups** | | | | | | | | | |
| --- | --- | --- | --- | --- | --- | --- | --- | --- | --- |
|  | | N | Mean | Std. Deviation | Std. Error | 95% Confidence Interval for Mean | | Minimum | Maximum |
|  |  |  |  |  |  | Lower Bound | Upper Bound |  |  |
| **Proportion of NCV** | **1.00** | 97 | .1099 | .11166 | .01134 | .0874 | .1324 | .00 | .64 |
|  | **2.00** | 296 | .0391 | .07653 | .00445 | .0304 | .0479 | .00 | .77 |
|  | **3.00** | 229 | .0788 | .10937 | .00723 | .0645 | .0930 | .00 | .77 |
|  | **4.00** | 118 | .0381 | .06963 | .00641 | .0254 | .0508 | .00 | .39 |
|  | **Total** | 740 | .0605 | .09541 | .00351 | .0536 | .0674 | .00 | .77 |
| **Proportion of FFV** | **1.00** | 97 | .2655 | .21338 | .02167 | .2225 | .3085 | .00 | .88 |
|  | **2.00** | 296 | .1095 | .16868 | .00980 | .0902 | .1288 | .00 | .75 |
|  | **3.00** | 229 | .1608 | .17668 | .01168 | .1378 | .1838 | .00 | .88 |
|  | **4.00** | 118 | .1163 | .17521 | .01613 | .0844 | .1483 | .00 | .78 |
|  | **Total** | 740 | .1469 | .18542 | .00682 | .1335 | .1603 | .00 | .88 |
| **Proportion of FV** | **1.00** | 97 | .1645 | .12374 | .01256 | .1396 | .1895 | .00 | .58 |
|  | **2.00** | 296 | .0915 | .12966 | .00754 | .0766 | .1063 | .00 | .72 |
|  | **3.00** | 229 | .1170 | .11016 | .00728 | .1027 | .1314 | .00 | .57 |
|  | **4.00** | 118 | .1126 | .16587 | .01527 | .0823 | .1428 | .00 | .86 |
|  | **Total** | 740 | .1123 | .13168 | .00484 | .1028 | .1218 | .00 | .86 |
| **Proportion of CV** | **1.00** | 97 | **.4788** | .43675 | .04435 | .3908 | .5668 | .00 | 2.78 |
|  | **2.00** | 296 | **.7599** | .33155 | .01927 | .7220 | .7979 | .00 | 1.00 |
|  | **3.00** | 229 | **.6434** | .34039 | .02249 | .5991 | .6877 | .00 | 1.00 |
|  | **4.00** | 118 | **.7324** | .34696 | .03194 | .6692 | .7957 | .00 | 1.00 |
|  | **Total** | 740 | **.6826** | .36358 | .01337 | .6564 | .7089 | .00 | 2.78 |
| **Proportion of non-CV** | **1.00** | 97 | **.5212** | .43675 | .04435 | .4332 | .6092 | .00 | 1.00 |
|  | **2.00** | 296 | **.2401** | .33155 | .01927 | .2021 | .2780 | .00 | 1.00 |
|  | **3.00** | 229 | **.3566** | .34039 | .02249 | .3123 | .4009 | .00 | 1.00 |
|  | **4.00** | 118 | **.2676** | .34696 | .03194 | .2043 | .3308 | .00 | 1.00 |
|  | **Total** | 740 | **.3174** | .36358 | .01337 | .2911 | .3436 | .00 | 1.00 |

**1 for T2DM, 2 for HTN,3 for T2DM and HTN ,4 for Control, non-CV=NCV+FFV+FV**

**5. Table S2**

| **Table S2 Tukey HSD multiple comparisons of Proportion of non-CV between groups** | | | | | | | |
| --- | --- | --- | --- | --- | --- | --- | --- |
|  | | | | | | | |
| Dependent Variable |  |  | Mean difference | Std. Error | **Sig.** | 95% Confidence Interval | |
|  |  |  |  |  |  | Lower Bound | Upper Bound |
| **Proportion of non-CV** | **1.00** | **2.00** | .28114^*^ | .04119 | **<.001** | .1751 | .3872 |
|  |  | **3.00** | .16458^*^ | .04265 | **<.001** | .0548 | .2744 |
|  |  | **4.00** | .25363^*^ | .04826 | **<.001** | .1294 | .3779 |
|  | **2.00** | **1.00** | -.28114^*^ | .04119 | **<.001** | -.3872 | -.1751 |
|  |  | **3.00** | -.11656^*^ | .03099 | **.001** | -.1963 | -.0368 |
|  |  | **4.00** | -.02751 | .03833 | **.890** | -.1262 | .0712 |
|  | **3.00** | **1.00** | -.16458^*^ | .04265 | **<.001** | -.2744 | -.0548 |
|  |  | **2.00** | .11656^*^ | .03099 | **.001** | .0368 | .1963 |
|  |  | **4.00** | .08904 | .03990 | **.116** | -.0137 | .1918 |
|  | **4.00** | **1.00** | -.25363^*^ | .04826 | **<.001** | -.3779 | -.1294 |
|  |  | **2.00** | .02751 | .03833 | **.890** | -.0712 | .1262 |
|  |  | **3.00** | -.08904 | .03990 | **.116** | -.1918 | .0137 |
| ***. The mean difference is significant at the 0.05 level. 1 for T2DM, 2 for HTN,3 for T2DM and HTN ,4 for Control, non-CV=NCV+FFV+FV** | | | | | | | |

6. **Table S3**

| **Table S3 Dunn-Bonferroni post-hoc test was used to determine NCV differences between groups** | | | | | | |
| --- | --- | --- | --- | --- | --- | --- |
|  |  | Mean difference | Std. Error | **Sig.** | 95% Confidence Interval | |
|  |  |  |  |  | Lower Bound | Upper Bound |
| **1.00** | **2.00** | 8.52113^*^ | 1.28039 | **<.001** | 5.1373 | 11.9050 |
|  | **3.00** | -2.29929 | 1.38381 | **.581** | -5.9565 | 1.3579 |
|  | **4.00** | 9.86224^*^ | 1.33566 | **<.001** | 6.3323 | 13.3922 |
| **2.00** | **1.00** | -8.52113^*^ | 1.28039 | **<.001** | -11.9050 | -5.1373 |
|  | **3.00** | -10.82042^*^ | 1.01682 | **<.001** | -13.5077 | -8.1331 |
|  | **4.00** | 1.34111 | .95025 | **.951** | -1.1703 | 3.8525 |
| **3.00** | **1.00** | 2.29929 | 1.38381 | **.581** | -1.3579 | 5.9565 |
|  | **2.00** | 10.82042^*^ | 1.01682 | **<.001** | 8.1331 | 13.5077 |
|  | **4.00** | 12.16153^*^ | 1.08558 | **<.001** | 9.2925 | 15.0306 |
| **4.00** | **1.00** | -9.86224^*^ | 1.33566 | **<.001** | -13.3922 | -6.3323 |
|  | **2.00** | -1.34111 | .95025 | **.951** | -3.8525 | 1.1703 |
|  | **3.00** | -12.16153^*^ | 1.08558 | **<.001** | -15.0306 | -9.2925 |
| ***. The mean difference is significant at the 0.05 level. 1 for T2DM, 2 for HTN,3 for T2DM and HTN ,4 for Control.** | | | | | | |
